# Prevalence of neurodevelopmental disorders and their impact on the health and social well-being among looked after children (LAC): a systematic review protocol

**DOI:** 10.1186/s13643-022-01923-6

**Published:** 2022-03-19

**Authors:** Nicola Heady, Alan Watkins, Ann John, Hayley Hutchings

**Affiliations:** Swansea University Medical School, Institute of Life Science 2, Floor 2, Singleton Campus, Swansea, SA2 8PP Wales

**Keywords:** Looked after child, Adopted, Residential, Kinship, Prevalence, Neurodevelopmental disorder, Health, Social, Well-being, Systematic review, Protocol

## Abstract

**Background:**

Looked after children (LAC) that are placed in either a foster, kinship, residential care setting or transition to adoption continue to develop debilitating disorders that significantly impact their overall health and social well-being. The prevalence of these disorders is often depicted under broad categories such as mental, behavioural or neurodevelopmental disorders (NDDs). Limited in research is the prevalence of what specific disorders fall under these broad categories. NDDs such as autism spectrum disorder (ASD) and attention deficit hyperactivity disorder (ADHD) which fall under an umbrella group in the expert field of genetics and neuropsychiatry will be explored. Unsupported, these disorders can lead to suboptimal health and social outcomes for both the child and family. In the general population, the prevalence of these NDDs and impacts on health and social well-being are relatively well documented, but for minority groups such as LAC, research is extremely limited. This review aims to estimate the prevalence of NDDs among LAC and explore how they might impact the health and social well-being of these vulnerable children. If feasible, the review will compare the prevalence rates to those children who are not looked after, to illuminate any differences or similarities between populations.

**Methods:**

PubMed, ASSIA, IBSS, Web of Science, PsychINFO, Scopus, Psych articles, Social Care Online, secondary, grey literature and government publications will be searched to identify any eligible studies. No restrictions will be placed on country, design or year of publication. Studies must provide primary data on the prevalence or incidence of NDDs for individuals < 25 years of age, supported by either a diagnostic code, standardised diagnostic assessment tool or survey response. The Joanna Briggs Institute (JBI) critical appraisal tools will be utilised to assess the quality and bias and the random-effects model used to estimate a pooled prevalence of NDDs.

**Discussion:**

Attaining an estimated prevalence of these NDDs and identifying any impacts on health and social well-being might inform key stakeholders in health, educational and social sectors with important information that might aid in the early identification and intervention to safeguard and meet the unique needs of these children.

**Systematic review registration:**

PROSPERO CRD4201913103.

## Background

The definition and terminology of a ‘looked after’ child differs on an international level. However, they all share commonality as they represent those children who have been removed from their biological home, involuntarily or voluntarily. The local authority or state then assume the first role of the collective ‘corporate parent’, responsible for caring, protecting and safeguarding the child [1–4]. These children may be placed in various short-term, long-term or fixed care settings where reunification with their biological parent/s is the primary objective. However, for varying reasons, reunification may be delayed or not always possible, and during this process, many children are placed in the care of wider family members, residential or foster care or transition into an adoptive setting [5, 6].

Although there is a myriad of reasons why children become looked after, in countries such as the United Kingdom (UK) and the United States of America (USA), the most prominent reasons for referral into services are abuse and neglect [4, 7]. Physical abuse, emotional abuse, sexual abuse, not meeting basic needs and emotional or medical neglect are just some of the complexities that underpin the broad term of ‘abuse and neglect’, while other reasons such as parental incarceration, death and abandonment contribute to referrals [4]. In Finland, abuse and neglect are the less common reasons for referral, and children are more often placed in a care setting due to parental physical/mental illness or a child’s special care/special educational needs [8].

Once placed in the care setting, the child becomes legally protected, and the collective ‘corporate parent’ who is made up of a multi-disciplinary group of public bodies and wider partner agencies take full responsibility for the safeguarding and care of the child [4]. Yet, despite being embedded in this multi-disciplinary group of professionals and services, research continues to depict a bleak future for these children predicting inauspicious health and social outcomes. Poor outcomes associated with mental health, disability, suicide, criminal system involvement, teenage parenthood, substance misuse and educational attainment have become the predicted ‘norm’ [9–15]. Contributory factors such as multifaceted, adverse childhood experiences (ACEs), psycho-social problems, poor socio-economic status, multiple residential and education moves and complexities in the consent process are just some of the dynamics that frequently underpin these outcomes and prohibit the child from the early intervention which impacts their overall health and social well-being [16–18].

Situated within these poor outcomes is the important area of mental health and associated mental disorders. The higher prevalence of mental disorders in LAC is well documented in research, often attributed to ACEs or poor socio-economic environments [9–12]. However, these prevalence studies are often depicted and categorised under broad headings such as mental, behavioural or neurodevelopmental [10, 12, 13]. Although significant findings, limited within secondary literature is a more in-depth, individual analysis of what specific disorders represent these broad categories.

Mental disorders can significantly affect an individuals’ mental and physical health, emotions, memory, ability to learn and socialise at some point in their life [19, 20]. Some may affect the individual on an intermittent basis or manifest during periods of immense stressful or challenging life events [19, 20]. However, NDDs are life-long and have the same detrimental impact on the individual while also creating challenges in the biological processes associated with both the brain and/or nervous system [19–26].

NDDs are a complex, multifaceted subject area of which we are only at the tip of exploration. They cross a wide clinical spectrum, and many are suggested to have genetic and hereditary origins [19–26]. Some disorders such as Prader-Willi syndrome or fragile X syndrome can be diagnosed with a medical test. However, many NDDs such as ASD or ADHD can only be diagnosed solely based on behaviour [27–30]. Furthermore, emerging research now ascertains that many of these NDDs frequently co-occur and co-exist but can take years to diagnose as they display similar symptomology in areas such as impaired social communication and interaction skills, similar sensory and motor dysfunctions, sleeping and eating difficulties, attachment issues and attention problems [31–34]. As a result, these similarities make early diagnosis even more challenging for the professional, and many do not attain a diagnosis until they reach adolescent or adult age [30, 35–37].

This is already reflected in LAC research and in the general population where many disorders are often misdiagnosed, over-diagnosed or completely missed [36, 38–40]. For the front-line professional who is primarily responsible for referring the LAC to appropriate services, research ascertains that many professionals address this area as challenging, creating potentially significant barriers for early intervention [41]. Difficult to diagnose, research ascertains that they can be often inadvertently overlooked by the clinician or possibly misdiagnosed as the urgency to treat or provide the child with services overrides further exploration of the underlying behavioural symptomology [40, 41]. This lack of early diagnosis and intervention can leave the child struggling in the home, school and social setting with no support or understanding, attempting to navigate a world that makes no sense to them. For the LAC who is already vulnerable and has already experienced complex ACEs, adding the complexity of an NDD could place these children at an even greater disadvantage.

## Rationale

This review will explore the prevalence and impact of NDDs that affect the brain and/or nervous system but cannot be medically tested for [19–26]. The disorders of interest for this review are foetal alcohol spectrum disorder (FASD), reactive attachment disorder (RAD), ADHD, bipolar, schizophrenia, obsessive-compulsive disorder (OCD), eating disorders, ASD (pervasive development disorder, Asperger’s), mathematics disorder (dyscalculia), intellectual disability, reading and writing disorder (dyslexia), speech and language impairment, social (pragmatic) communication disorder, tic disorder and stereotypic movement disorder (dyspraxia) [42].

Although the majority of these NDDs are categorised as NDDs in the Diagnostic and Statistical Manual of Mental Disorders (DSM 5) and International Classification of Diseases (ICD 11), some are still classified under the broad heading of mental disorders. Nevertheless, they all share a commonality affecting the development of the brain and/or nervous system. As the nosology of psychiatry continues to evolve, many of these NDDs fall under new categories as is in the instance with ADHD now being included in the ICD 11 as a NDD, and many have been grouped and labelled as NDDs in the literature, by leading experts in the areas of genetics and neuropsychiatry [19–26].

These manuals which significantly influence the clinical sector, are the most up to date and authoritative guidelines for professionals and research. Yet, it is also common knowledge that the DSM and ICD manuals can take over 10 years to be updated [42, 43]. Therefore, the review was concerned it might under-identify the prevalence rates of NDDs in this vulnerable population. As an example, ADHD was classified as a disruptive, behavioural disorder for decades in previous manuals and autism spectrum disorder as ‘childhood schizophrenia’ [44–46]. If this review had been conducted prior to these updates, research might have missed important information and not included these NDDs in the inclusion criteria.

To date, there has been limited research applied to NDDs in respect of LAC. They have often been noted in LAC research but under researched and omitted from results or not elaborated upon for varying reasons, such as low sample numbers [47–49]. Nevertheless, new increased awareness of the diversity of how these disorders manifest in the child is rapidly increasing in the psychiatric field with diagnostic assessments and accuracy improving [28–30]. A recent systematic review by Willis et al. found that ADHD was higher in LAC compared to non-LAC [50]. Although a significant finding, the review acknowledges that the results should be interpreted with some caution as ADHD shares similar behavioural symptomology with other conditions [30, 51]. While early diagnosis and intervention are important, overdiagnosis and misdiagnosis can be just as detrimental to the child and have serious implications on what specific support and interventions they receive [52]. Furthermore, the review used a national prevalence from each country of study to compare the rates. This review will extract the true prevalence rates from the included studies and include two further groups of interest: children placed in a kinship setting and adopted children [50].

The literature relating to the impacts on health and social well-being associated with having an NDD in the general population is still limited but relatively well documented. Research proposes that early identification and intervention are key components that improve the future health and social well-being of children with NDDs [35–37, 53–60]. Nevertheless unsupported, many children frequently experience a detrimental impact on their mental health, physical health and overall social well-being which frequently leads to poor mental health, debilitating anxiety, depression, sexual abuse, self-harm, suicide, eating disorders, family breakdown, low educational attainment and potential exclusion from education, employment and society [35–37, 53–60].

Extremely limited in research are the impacts on the health and social well-being of LAC who have an NDD. Safeguarding these children remains a pivotal and paramount part of health and social services. If we incorporate a NDD into the complexities that encompass these children’s lives, it is hypothesised that there might be further issues with safeguarding as a result of their impairments and challenges that are often associated with these disorders [61–63]. These lifelong disorders require proactive planning for specific, specialised support throughout education, health and social care settings. Accurate, early identification and specialised interventions designed to improve future health and social well-being can only reduce future demands into services but, more importantly, improve the overall well-being of these vulnerable children to flourish within society and lead a fulfilling life.

## Objectives

Rates of children entering the care system are increasing on a global level. The demands on services are placing immense pressures on government funding, third sectors, front-line services, families and more importantly on the children themselves [64]. The review proposes it is an appropriate time to estimate the prevalence of NDDs in the LAC population and investigate the impacts on the health and social well-being of these children. The review objectives are to:

1. Estimate the prevalence of NDDs in looked after children and if feasible compare with those children who are not looked after
2. Identify what impacts NDDs may have on the health and social well-being of the looked after child

## Methods

This protocol has been registered with the PROSPERO database for systematic reviews (registration number: CRD4201913103) and will be reported in accordance with the guidelines outlined in the PRISMA [65].

### Search strategy

The PICO approach has been used to help define the primary research questions and formulate the search strategy: (P) Population: looked after children; (I) Phenomenon of interest and outcome: prevalence and impact on health and social well-being; (C) Condition: neurodevelopmental disorders.

‘What is the prevalence of NDDs and how do they impact the health and social well-being of the looked after child?’

Synonyms will be used to expand the results due to the extensive array of terminology that is often associated with both LAC and the NDDs being explored (Table 1). This is demonstrated in an example search strategy (see the “Table 2 in Appendix”). The following databases will be searched to identify relevant primary literature for the review: PubMed, ASSIA, IBSS, Web of Science, PsychINFO, Scopus, Psych articles and Social Care Online. The proposed databases were selected based on those identified in other peer-reviewed studies that explored similar outcomes of interest [66–68]. The review will additionally systematically hand search published and unpublished secondary, grey and governmental literature as they can be a rich information source for exploring citations and reference lists to further identify any new primary articles and potentially alleviate publication bias [69].

**Table 1:** Neurodevelopmental disorders and associated synonyms

Disorder	Other synonyms
Schizophrenia	
Bipolar disorder	Paediatric bipolar disorder
Social phobia, unspecified	Social anxiety or social anxiety phobia
Obsessive-compulsive disorder	OCD
Eating disorders, unspecified	Bulimia or anorexia
Mild intellectual disability	Developmental academic disorder or learning difficulties
Social pragmatic communication disorder	Social communication disorder
Developmental disorder of speech and language, unspecified	Speech and language disorder
Specific reading disorder	Reading disorder or written disorder or dyslexia
Mathematics disorder	Acalculia or mathematic disability or dyscalculia
Specific developmental disorder of motor function	Developmental coordination disorder or dyspraxia
Autistic disorders	Autism spectrum disorder, pervasive development disorder, ASD and (including Asperger’s syndrome)
Attention deficit hyperactivity disorder	ADHD
Reactive attachment disorder	RAD
Tic disorder	Tourette’s syndrome
Stereotyped movement disorder	
Foetal alcohol syndrome	Foetal alcohol spectrum disorder or FASD

### Eligibility criteria

No restriction will be placed on country, design or year of publication. Only publications in the English language will be included and filters such as language limits applied if feasible. Databases that have distinctive processes that require adaptation in searching tools will be guided and supervised by the residing university librarians to ensure consistency.

Although the review acknowledges that the legal definition and terminology for a LAC can vary on an international level, many of these countries share commonality with the UK legal definition of a LAC which derives from both the Children Act 1989 and the Children Act 1995, Scotland [1–4]. A LAC is predominantly a child who can be accommodated under a voluntary agreement with their parents, who is the subject of a care order or interim care order, the subject of emergency orders for their protection, who is compulsorily accommodated. This can include children remanded or subject to a criminal justice supervision order with a residence requirement and children who are subject to a valid permanence order under section 80 of the Adoption and Children (Scotland) Act 2007 [1–4]. Any limitations in varying international terminology will be acknowledged and addressed in the results.

For this review, the term LAC will represent those children who have been removed voluntarily or involuntarily from their biological home and placed in an ‘out of home’ care setting in either residential, foster or kinship including adoption [70]. Although the adopted child is no longer considered a LAC after completing the adoption process, a large majority of these children would have previously been LAC [71, 72]. The adoption can also be reverted if the relationship breaks down or the parent wishes to reclaim the child during the process [71, 72]. To address this possible limitation, a distinction will be made in the results to acknowledge any difference in care profiles, and a sensitivity analysis will be conducted to differentiate the prevalence rates between the groups.

Non-LAC will represent those children who are not placed in a care setting or have never been placed in a care setting. This will also include children who access respite care, often referred to as a ‘child in need’ or a ‘child in protection’ as they have not been removed from the biological home and remain with their biological parent/s [4].

There are limitations to this which will be addressed in the review as the child may be reunified with their biological parent/s or have re-entered the care setting pre or prior to when the study collated the data [70]. However, reunification is not always sustained as a result of varying factors such as poor parental physical and mental health and continuous substance misuse abuse [5, 6].

As there is a duty of care to support some of these children up to the age of 25 in the UK, this will be the upper limit to the inclusion criteria for this review. This will enable the review to capture those individuals who might have been diagnosed later in their life with these NDDs. Additionally, this time period will enable the review to explore the earlier impacts on the health and social well-being of these children, as a result of having an NDD for earlier intervention purposes [70].

As the review aims to attain a lifetime prevalence, studies must provide primary data on prevalence or incidence of NDDs for individuals < 25 years of age, supported by either a diagnostic code, standardised diagnostic assessment tool or survey response. There are limitations to this approach with varying factors such as self-reporting bias, coding of disorders applied without clinician interviews and diagnostic codes changing over time for some of these disorders [38]. However, the review will follow other studies of similar methodology and include in the collated characteristics the description of the tools, codes and diagnostic manual used for transparency purposes. The review will also acknowledge any limitations and address them in the review.

With the introduction of the new International Classification of Diseases 11th Revision (ICD-11) guidelines which acknowledge that many of these NDD disorders now co-exist; this review will include studies that have prevalence rates for children who have more than one diagnosis [73].

Health and social well-being in its purest sense are about maintaining good physical and mental health which integrates with safeguarding, social interaction, maintaining healthy relationships and enabling individuals to bridge social networks to increase social mobility [74]. Although it is anticipated that the health and social outcomes may reflect those that are already associated with those children who have an NDD in the general population, it was agreed that predefining impacts or outcomes might prohibit or exclude important information due to the limited amount of available studies with regard to these vulnerable children and the specific associated disorders being explored.

### Study selection

The study selection will be undertaken by three reviewers. Initially, all studies will be screened by review of title then followed by review of abstract. The next stage will be to apply the exclusion and inclusion criteria and remove studies that do not fit the specified criterion. Following this, all remaining papers will be fully screened.

As the study is exploring two areas of interest, the first stage will appraise the articles that detail the prevalence of NDDs in the LAC population and if feasible compare to prevalence rates associated with non-LAC. The second stage will appraise any articles that detail the impacts on the health and social well-being of LAC who have these NDDs.

Intermittent searches will be conducted while the review is being undertaken to ensure that new literature is not missed [75]. A reviewer’s meeting will be scheduled to seek consensus and to agree if any more primary studies should be included in the review, to aid in addressing the research questions.

### Data collection and extraction

An extraction form will be designed to examine and collate the information related to the areas of interest. Characteristics such as study name, country, total sample size, age, gender (% male), type of placement, case ascertainment method, NDD, diagnostic system used, diagnostic instrument, number of cases of neurodevelopmental disorders and any impacts on health and social well-being will be detailed in a table format. The form will provide a clear framework for collating the data [75].

### Quality and bias assessment

Adopting similar methods used in other studies, the Joanna Briggs Institute (JBI) critical appraisal tools, will be utilised [76]. These are appropriate tools that have been used in other reviews and suggested to be applicable due to the diversity of the designs normally anticipated in a systematic review. All appraisal tools will address the bias in design, conduct and analysis [76]. For consistency purposes, two reviewers will independently assess and appraise the studies [75]. If there is a divergence in assessment, a review meeting will be arranged and the third reviewer will become a mediator to reach a consensus. This will be documented within the review as a narrative summary, to provide clarity and transparency.

### Data synthesis and analysis

Studies that detail primary data for both LAC and non-LAC will be used to conduct a meta-analysis to illuminate any differences or similarities between populations. If a meta-analysis is not feasible, articles that detail prevalence data of NDDs in LAC will be pooled together and represented in forest plots [75].

It is anticipated that there may be high heterogeneity between studies; therefore, the random effect model will be used to estimate a mean of a distribution of effects [75, 77]. The effect will be either be expressed as odds ratio or relative risk. The measure will depend on whether meaningful incidences or prevalence is available due to the anticipated differences in research design for the included studies [78].

Forest plots will be used to provide a graphical representation of the results. Funnel plots will be considered for exploring heterogeneity and possible publication bias provided there are sufficient studies [75, 77]. However, the review acknowledges that funnel plot asymmetry can occur for many other varying reasons, and therefore, if required appropriate, tests will be used to further explore [79, 80]. Subsequently, where statistical analysis is not possible, a descriptive analysis will be provided detailing the prevalence ranges of the NDDs (e.g. ADHD ranging from 2 to 16% in six of the studies).

To evaluate the impacts on health and social well-being, both qualitative and quantitative data will be analysed to enable a descriptive framework to occur. All information relating to the impacts on the health and social well-being of this population, as a result of having these NDDs, will be analysed by two reviewers. In the final stage, all analyses will be amalgamated to provide a discussion of the results attained.

### Software considerations

The SUMARI software package, a comprehensive review management system that has been designed to assist researchers in the health and social sciences to conduct and support systematic reviews will be utilised to extract, critically appraise and part analyse the data [81]. To conduct the meta-analysis, the RevMan 5.0 software package will be used to meet the needs of the research design.

### Dissemination and research integrity

The findings will be disseminated through various pathways, such as peer review journals, public and third sector organisations, Welsh government policy departments, the Children’s Commissioner, appropriate paediatric National and International Conferences, using various methods such as posters, websites and presentations.

### Limitations

The protocol anticipates that there will be limitations to acknowledge, due to the complexities that surround this population. One limitation will be the definition and terminology utilised for LAC in varying countries. There will be differences in profiles that determine the definition of a LAC or non-LAC, which will be noted and addressed in the review should it impact the results in any way.

Secondly, the review acknowledges that language bias can often occur as a result of only using articles in the English language. The review acknowledges that this may exclude some important articles and lead to an incomplete, accurate representation of the evidence [82].

It is also acknowledged that diagnostic coding will have evolved and changed in relation to the classification of these disorders outlined in the previous and current ICD and DSM. As an example, ASD will have previously included those individuals diagnosed with ‘Asperger’s’. Therefore, diagnostic coding will be scrutinised to ensure that the prevalence rates include the evolved terminology associated with the NDD.

Lastly, a limitation for making the upper age limit < 25 may exclude older individuals who have previously been LAC or non-LAC diagnosed with or without these disorders. Additional adverse life experiences after leaving the care setting are often associated with this population [70]. Therefore, going any higher in age although a significant knowledge gap in research could confound the results of the review.

## Discussion

Willis et al. conducted a significant piece of research and found that the prevalence of ADHD was much higher in LAC, and this review aims to contribute to this existing research [50]. However, this is the first systematic review to our knowledge that has focused on a wider group of specific NDDs. Although this review will contribute to understanding the prevalence of these NDDs in this population, the results must be interpreted with caution. There are varying factors that could either inflate or deflate prevalence rates. If children are frequently being misdiagnosed, over-diagnosed or missed altogether in the general population, one must also consider that this may be occurring in relation to these vulnerable children [41, 83–86]. Unravelling their complex social, emotional and behavioural symptomology coupled with expected behaviours associated with trauma as a result of ACEs can only create confusion and uncertainty for many front-line professionals and clinicians.

Nevertheless, estimating the prevalence of NDDs and understanding the impacts on the health and social well-being of LAC can only enhance and contribute to existing LAC literature. This review has the capacity to narrow the research focus on specific disorders and further explore why the disorders may be more prevalent or not or impede on these vulnerable children. From an economic and social mobility perspective, the results might also inform the key stakeholders of where to direct earlier appropriate services that may be required in health, educational and social sectors to meet the unique needs of these children. An earlier intervention designed to improve future health and social well-being can only reduce future demands for these services. From a preventative and safeguarding viewpoint, it is of great importance that we explore if these NDDs place these already vulnerable children at even greater risk in society.

## Data Availability

Not applicable.

## Appendix

Table 2

**Table 2:** Search strategy from ProQuest 10 June 2019, 10:11

Set#	Searched for	Databases	Results
S1	(((MAINSUBJECT.EXACT(“Hoarding disorder”) OR MAINSUBJECT.EXACT(“Emotional disorders”) OR MAINSUBJECT.EXACT(“Obsessive compulsive disorder”) OR MAINSUBJECT.EXACT(“Psychosis”) OR MAINSUBJECT.EXACT(“Mental depression”) OR MAINSUBJECT.EXACT(“Bulimia”) OR MAINSUBJECT.EXACT(“Eating disorders”) OR MAINSUBJECT.EXACT(“Family medical history”) OR MAINSUBJECT.EXACT(“Behavior disorders”) OR MAINSUBJECT.EXACT(“Bipolar disorder”) OR MAINSUBJECT.EXACT(“Personality disorders”)) OR (MAINSUBJECT.EXACT(“Obsessive compulsive disorder”) OR MAINSUBJECT.EXACT(“Psychosis”) OR MAINSUBJECT.EXACT(“Mental depression”) OR MAINSUBJECT.EXACT(“Bulimia”) OR MAINSUBJECT.EXACT(“Eating disorders”) OR MAINSUBJECT.EXACT(“Family medical history”) OR MAINSUBJECT.EXACT(“Behavior disorders”) OR MAINSUBJECT.EXACT(“Bipolar disorder”) OR MAINSUBJECT.EXACT(“Personality disorders”))) AND la.exact(“English”)) AND PEER(yes)	International Bibliography of the Social Sciences (IBSS)	7052
S2	((ti(mental health OR mental disorder* OR mental retardation OR psychiatric disorder* OR psycholog* disorder OR psycholog* disability*) OR ab(mental health OR mental disorder* OR mental retardation OR psychiatric disorder* OR psycholog* disorder OR psycholog* disability*)) AND la.exact(“English”)) AND PEER(yes)	International Bibliography of the Social Sciences (IBSS)	20,350
S3	((ti(development* disorder* OR development* disability* OR, neurodevelop* disorder* OR learning disability OR learning disorder) OR ab(development* disorder* OR development* disability* OR, neurodevelop* disorder* OR learning disability OR learning disorder)) AND la.exact(“English”)) AND PEER(yes)	International Bibliography of the Social Sciences (IBSS)	3936
S4	((ti(foetal Alcohol Spectrum Disorder* OR FASD OR Reactive Attachment Disorder OR RAD OR Attention Deficit Hyper* Disorder OR ADHD OR paediatric bipolar OR bipolar disorder OR schizophrenia OR Obsessive Compulsive Disorder OR OCD OR Eating Disorders OR Bulimia OR bulimia OR bulimic OR anorexia OR anorexic OR Autis* OR Autism Spectrum Disorder OR ASD OR Pervasive Development Disorder OR Asperger* OR Specific Language Disability OR SLD OR Speech and Language Impairment OR Mathematics Disorder OR mathematic disability OR Dyscalculia OR Intellectual Disability OR Reading disorder OR written Disorder OR Dyslexia OR Social Communication Disorder OR social pragmatic language OR Tic Disorder OR Stereotypic Movement Disorder OR Developmental coordination disorder OR Dyspraxia OR Social Anxiety OR social anxiety phobia) OR ab(foetal Alcohol Spectrum Disorder* OR FASD OR Reactive Attachment Disorder OR RAD OR Attention Deficit Hyper* Disorder OR ADHD OR paediatric bipolar OR bipolar disorder OR schizophrenia OR Obsessive Compulsive Disorder OR OCD OR Eating Disorders OR Bulimia OR bulimia OR bulimic OR anorexia OR anorexic OR Autis* OR Autism Spectrum Disorder OR ASD OR Pervasive Development Disorder OR Asperger* OR Specific Language Disability OR SLD OR Speech and Language Impairment OR Mathematics Disorder OR mathematic disability OR Dyscalculia OR Intellectual Disability OR Reading disorder OR written Disorder OR Dyslexia OR Social Communication Disorder OR social pragmatic language OR Tic Disorder OR Stereotypic Movement Disorder OR Developmental coordination disorder OR Dyspraxia OR Social Anxiety OR social anxiety phobia)) AND la.exact(“English”)) AND PEER(yes)	International Bibliography of the Social Sciences (IBSS)	10,122
S5	S1 OR S2 OR S3 OR S4	International Bibliography of the Social Sciences (IBSS) These databases are searched for part of your query.	34,993
S6	((ti(Looked after child* OR LAC OR foster child* OR foster care OR out of home care OR out-of-home care OR residential care OR kinship care OR adopt* OR child* in secure unit OR child* in welfare OR child* in protection) OR ab(Looked after child* OR LAC OR foster child* OR foster care OR out of home care OR out-of-home care OR residential care OR kinship care OR adopt* OR child* in secure unit OR child* in welfare OR child* in protection)) AND la.exact(“English”)) AND PEER(yes)	International Bibliography of the Social Sciences (IBSS)	88,130
S7	S5 AND S6	International Bibliography of the Social Sciences (IBSS) These databases are searched for part of your query.	1649
S8	((ti(prevalence OR incidence OR occurrence* OR rate* OR frequency) OR ab(prevalence OR incidence OR occurrence* OR rate* OR frequency)) AND la.exact(“English”)) AND PEER(yes)	International Bibliography of the Social Sciences (IBSS)	187,899
S9	S7 AND S8	International Bibliography of the Social Sciences (IBSS) These databases are searched for part of your query.	278

## Abbreviations

LAC: Looked after child/children
ICD: International Classification of Diseases
DSM: Diagnostic and Statistical Manual of Mental Disorders

## References

[1] Courtney ME (2009). The difficult transition to adulthood for foster youth in the US: implications for the state as corporate parent and commentaries. Soc Policy Rep.

[2] Mendes P, Michell D, Wilson JZ, SANDERS R. (2014). Young people transitioning from out-of-home care and access to higher education: a critical review of the literature. Child Aust.

[3] Department of Education (2018). Promoting the education of looked-after and previously looked-after children.

[4] Royal College of Paediatrics and Child Health (RCPCH) ‘Looked after children (LAC) guidance’ Accessed 11th May 2021 at: https://www.rcpch.ac.uk/resources/looked-after-children-lac

[5] Carlson L, Hutton S, Priest H, Melia Y (2020). Reunification of looked-after children with their birth parents in the United Kingdom: a literature review and thematic synthesis. Child Fam Soc Work.

[6] Fernandez E, Delfabbro P, Ramia I, Kovacs S (2019). Children returning from care: the challenging circumstances of parents in poverty. Child Youth Serv Rev.

[7] Clark SL, Palmer AN, Akin BA, Dunkerley S, Brook J (2020). Investigating the relationship between trauma symptoms and placement instability. Child Abuse Negl.

[8] Côté SM, Orri M, Marttila M, Ristikari T (2018). Out-of-home placement in early childhood and psychiatric diagnoses and criminal convictions in young adulthood: a population-based propensity score-matched study. Lancet Child Adoles Health.

[9] Ford T, Vostanis P, Meltzer H, Goodman R (2007). Psychiatric disorder among British children looked after by local authorities: comparison with children living in private households. Br J Psychiatry.

[10] Meltzer H, Gatward R, Corbin T, Goodman R, Ford T (2003). The mental health of young people looked after by local authorities in England.

[11] Lightfoot E, Hill K, LaLiberte T (2011). Prevalence of children with disabilities in the child welfare system and out of home placement: an examination of administrative records. Child Youth Serv Rev.

[12] Hill L, Baker C, Kelly B, Dowling S (2017). Being counted? Examining the prevalence of looked-after disabled children and young people across the UK. Child Fam Soc Work.

[13] NHS (2018) ‘Mental health of children and young people in England, 2017 [PAS]’ Accessed 3rd March at: https://digital.nhs.uk/data-and-information/publications/statistical/mental-health-of-children-and-young-people-in-england/2017/2017#key-facts

[14] Teyhan A, Wijedasa D, Macleod J (2018). Adult psychosocial outcomes of men and women who were looked-after or adopted as children: prospective observational study. BMJ Open.

[15] Blake AJ, Ruderman M, Waterman JM, Langley AK. Long-term effects of pre-adoptive risk on emotional and behavioral functioning in children adopted from foster care. Child Abuse Negl. 2021;1-13. 10.1016/j.chiabu.2021.105031 33757644

[16] Sanders J, Munford R, Liebenberg L, Ungar M (2014). Multiple service use: the impact of consistency in service quality for vulnerable youth. Child Abuse Negl.

[17] Morris L, Salkovskis P, Adams J, Lister A, Meiser-Stedman R (2015). Screening for post-traumatic stress symptoms in looked after children. J Children’s Serv.

[18] Häggman-Laitila A, Salokekkilä P, Karki S (2018). Transition to adult life of young people leaving foster care: a qualitative systematic review. Child Youth Serv Rev.

[19] Saarni SI, Suvisaari J, Sintonen H, Pirkola S, Koskinen S, Aromaa A (2007). Impact of psychiatric disorders on health-related quality of life: general population survey. Br J Psychiatry.

[20] Masson M, East-Richard C, Cellard C (2016). A meta-analysis on the impact of psychiatric disorders and maltreatment on cognition. Neuropsychology..

[21] D'Souza H, Karmiloff-Smith A (2017). Neurodevelopmental disorders. Wiley Interdiscip Rev Cogn Sci.

[22] Schork AJ, Won H, Appadurai V, Nudel R, Gandal M, Delaneau O, Revsbech Christiansen M, Hougaard DM, Bækved-Hansen M, Bybjerg-Grauholm J, Giørtz Pedersen M. A genome-wide association study of shared risk across psychiatric disorders implicates gene regulation during fetal neurodevelopment. Nat Neurosci. 2019;22(3):353–61. 10.1038/s41593-018-0320-0 PMC6497521 30692689

[23] Lo MT, Hinds DA, Tung JY, Franz C, Fan CC, Wang Y (2017). Genome-wide analyses for personality traits identify six genomic loci and show correlations with psychiatric disorders. Nat Genet.

[24] Thapar A, Cooper M, Rutter M (2017). Neurodevelopmental disorders. Lancet Psychiatry.

[25] Lichtenstein P, Carlström E, Råstam M, Gillberg C, Anckarsäter H (2010). The genetics of autism spectrum disorders and related neuropsychiatric disorders in childhood. Am J Psychiatr.

[26] Mitchell RH, Goldstein BI (2014). Inflammation in children and adolescents with neuropsychiatric disorders: a systematic review. J Am Acad Child Adolesc Psychiatry.

[27] Wolff JJ, Hazlett HC, Lightbody AA, Reiss AL, Piven J (2013). Repetitive and self-injurious behaviors: associations with caudate volume in autism and fragile X syndrome. J Neurodev Disord.

[28] Dimitropoulos A, Ho A, Feldman B (2013). Social responsiveness and competence in Prader-Willi syndrome: direct comparison to autism spectrum disorder. J Autism Dev Disord.

[29] Guthrie W, Swineford LB, Nottke C, Wetherby AM (2013). Early diagnosis of autism spectrum disorder: stability and change in clinical diagnosis and symptom presentation. J Child Psychol Psychiatry.

[30] Madsen KB, Ravn MH, Arnfred J, Olsen J, Rask CU, Obel C (2018). Characteristics of undiagnosed children with parent-reported ADHD behaviour. Eur Child Adolesc Psychiatry.

[31] Jang J, Matson JL, Williams LW, Tureck K, Goldin RL, Cervantes PE (2013). Rates of comorbid symptoms in children with ASD, ADHD, and comorbid ASD and ADHD. Res Dev Disabil.

[32] Panagiotidi M, Overton PG, Stafford T (2019). Co-occurrence of ASD and ADHD traits in an adult population. J Atten Disord.

[33] Hirjak D, Meyer-Lindenberg A, Fritze S, Sambataro F, Kubera KM, Wolf RC (2018). Motor dysfunction as research domain across bipolar, obsessive-compulsive and neurodevelopmental disorders. Neurosci Biobehav Rev.

[34] Emmons P, Anderson L. Understanding sensory dysfunction: learning, development and sensory dysfunction in autism spectrum disorders, ADHD, learning disabilities and bipolar disorder: Jessica Kingsley Publishers; 2005.

[35] Tierney S, Burns J, Kilbey E (2016). Looking behind the mask: social coping strategies of girls on the autistic spectrum. Res Autism Spectr Disord.

[36] Bargiela S, Steward R, Mandy W (2016). The experiences of late-diagnosed women with autism spectrum conditions: an investigation of the female autism phenotype. J Autism Dev Disord.

[37] Salim E, Fleming M, MacKay DF, Henderson A, Kinnear D, Clark D (2019). Neurodevelopmental multimorbidity and educational outcomes of 766,244 Scottish schoolchildren: Michael Fleming. Eur J Pub Health.

[38] Merten EC, Cwik JC, Margraf J, Schneider S (2017). Overdiagnosis of mental disorders in children and adolescents (in developed countries). Child Adolesc Psychiatry Ment Health.

[39] Klein B, Damiani-Taraba G, Koster A, Campbell J, Scholz C (2015). Diagnosing attention-deficit hyperactivity disorder (ADHD) in children involved with child protection services: are current diagnostic guidelines acceptable for vulnerable populations?. Child Care Health Dev.

[40] Chasnoff IJ, Wells AM, King L (2015). Misdiagnosis and missed diagnoses in foster and adopted children with prenatal alcohol exposure. Pediatrics..

[41] Pritchett R, Hockaday H, Anderson B, Davidson C, Gillberg C, Minnis H. Challenges of assessing maltreated children coming into foster care. Sci World J. 2016;2016:1–9. 10.1155/2016/5986835 PMC4736566 26881270

[42] ICD 10 (2018) ICD 10 Data ‘Mental, behavioural and neurodevelopmental disorders F01-F99’. Accessed 3 Mar 2019 at: https://www.icd10data.com/ICD10CM/Codes/F01-F99

[43] American Psychiatric Association. (2016). Diagnostic and Statistical Manual of Mental Disorders (DSM-5). Accessed 14 Jan 2021 at: https://www.psychiatry.org/psychiatrists/practice/dsm

[44] Harris JC (2014). New classification for neurodevelopmental disorders in DSM-5. Curr Opin Psych.

[45] Stein DJ, Szatmari P, Gaebel W, Berk M, Vieta E, Maj M (2020). Mental, behavioral and neurodevelopmental disorders in the ICD-11: an international perspective on key changes and controversies. BMC Med.

[46] Kulage KM, Goldberg J, Usseglio J, Romero D, Bain JM, Smaldone AM (2020). How has DSM-5 affected autism diagnosis? A 5-year follow-up systematic literature review and meta-analysis. J Autism Dev Disord.

[47] Kääriälä A, Hiilamo H (2017). Children in out-of-home care as young adults: a systematic review of outcomes in the Nordic countries. Child Youth Serv Rev.

[48] O’Higgins A, Sebba J, Luke N. What is the relationship between being in care and the educational outcomes of children. An international systematic review: University of Oxford Department of Education/University of Bristol; 2015.

[49] Sebba J, Berridge D, Luke N, Fletcher J, Bell K, Strand S, et al. The educational progress of looked after children in England: linking care and educational data: University of Oxford Department of Education/University of Bristol; 2015.

[50] Willis R, Dhakras S, Cortese S (2017). Attention-deficit/hyperactivity disorder in looked-after children: a systematic review of the literature. Curr Dev Disord Rep.

[51] Follan M, Anderson S, Huline-Dickens S, Lidstone E, Young D, Brown G (2011). Discrimination between attention deficit hyperactivity disorder and reactive attachment disorder in school aged children. Res Dev Disabil.

[52] González RA, Vélez-Pastrana MC, McCrory E, Kallis C, Aguila J, Canino G (2019). Evidence of concurrent and prospective associations between early maltreatment and ADHD through childhood and adolescence. Soc Psychiatry Psychiatr Epidemiol.

[53] Young S, González RA, Mullens H, Mutch L, Malet-Lambert I, Gudjonsson GH (2018). Neurodevelopmental disorders in prison inmates: comorbidity and combined associations with psychiatric symptoms and behavioural disturbance. Psychiatry Res.

[54] Hirvikoski T, Mittendorfer-Rutz E, Boman M, Larsson H, Lichtenstein P, Bölte S (2016). Premature mortality in autism spectrum disorder. Br J Psychiatry.

[55] Hakulinen C, Musliner KL, Agerbo E (2019). Bipolar disorder and depression in early adulthood and long-term employment, income, and educational attainment: a nationwide cohort study of 2,390,127 individuals. Depress Anxiety.

[56] Escott-Price V, Bracher-Smith M, Menzies G, Walters J, Kirov G, Owen MJ (2020). Genetic liability to schizophrenia is negatively associated with educational attainment in UK biobank. Mol Psychiatry.

[57] Russell AE, Ford T, Russell G (2015). Socioeconomic associations with ADHD: findings from a mediation analysis. PLoS One.

[58] Brede J, Remington A, Kenny L, Warren K, Pellicano E (2017). Excluded from school: autistic students’ experiences of school exclusion and subsequent re-integration into school. Autism Dev Lang Impairments.

[59] Kirby AV, Bakian AV, Zhang Y, Bilder DA, Keeshin BR, Coon H (2019). A 20-year study of suicide death in a statewide autism population. Autism Res.

[60] Lan WH, Bai YM, Hsu JW, Huang KL, Su TP, Li CT (2015). Comorbidity of ADHD and suicide attempts among adolescents and young adults with bipolar disorder: a nationwide longitudinal study. J Affect Disord.

[61] Heslop P, McAnelly S, Wilcockson J, Newbold Y, Avantaggiato-Quinn M, Meredith C. Do parents and carers experiencing violent and challenging behaviour from their children fit with safeguarding models of support? Messages from a Facebook study. J Adult Protection. 2019;21(6):285–95.

[62] Jeffs J, Cubbin S, Xenitidis K (2012). Adult attention-deficit hyperactivity disorder and child protection: review of evidence and child safeguarding guidelines. Psychiatrist.

[63] Fuller-Thomson E, Lewis DA, Agbeyaka SK (2016). Attention-deficit/hyperactivity disorder casts a long shadow: findings from a population-based study of adult women with self-reported ADHD. Child Care Health Dev.

[64] Crea TM, Lopez A, Taylor T, Underwood D (2017). Unaccompanied migrant children in the United States: predictors of placement stability in long term foster care. Child Youth Serv Rev.

[65] Page MJ, Moher D, Bossuyt PM, Boutron I, Hoffmann TC, Mulrow CD, et al. PRISMA 2020 explanation and elaboration: updated guidance and exemplars for reporting systematic reviews. BMJ. 2021;372:1–35. 10.1136/bmj.n160 PMC8005925 33781993

[66] Gough D, Thomas J, Oliver S (2012). Clarifying differences between review designs and methods. Systematic Rev.

[67] Xu Y, Bright CL (2018). Children’s mental health and its predictors in kinship and non-kinship foster care: a systematic review. Child Youth Serv Rev.

[68] Bronsard G, Alessandrini M, Fond G, Loundou A, Auquier P, Tordjman S, et al. The prevalence of mental disorders among children and adolescents in the child welfare system: a systematic review and meta-analysis. Medicine. 2016;95(7):1–17. 10.1097/MD.0000000000002622 PMC4998603 26886603

[69] Gough D, Richardson M. Systematic reviews. In: Advanced research methods for applied psychology: Routledge; 2018. p. 75–87.

[70] Jedwab M, Shaw TV (2017). Predictors of reentry into the foster care system: comparison of children with and without previous removal experience. Child Youth Serv Rev.

[71] Palacios J, Rolock N, Selwyn J, Barbosa-Ducharne M (2019). Adoption breakdown: concept, research, and implications. Res Soc Work Pract.

[72] Paniagua C, Palacios J, Jiménez-Morago JM (2019). Adoption breakdown and adolescence. Child Fam Soc Work.

[73] First MB, Reed GM, Hyman SE, Saxena S (2015). The development of the ICD-11 clinical descriptions and diagnostic guidelines for mental and behavioural disorders. World Psychiatry.

[74] Silver H. The contexts of social inclusion. Available at SSRN 2641272. 2015 Aug 8.

[75] Bashir Y, Conlon KC (2018). Step by step guide to do a systematic review and meta-analysis for medical professionals. Irish J Med Sci (1971-).

[76] Munn Z, Moola S, Riitano D, Lisy K (2014). The development of a critical appraisal tool for use in systematic reviews addressing questions of prevalence. Int J Health Policy Manag.

[77] Ward MM (2013). 2013. Estimating disease prevalence and incidence using administrative data: some assembly required. J Rheumatol.

[78] Schmidt CO, Kohlmann T (2008). When to use the odds ratio or the relative risk?. Int J Publi Health.

[79] Rücker G, Schwarzer G, Carpenter J (2008). Arcsine test for publication bias in meta-analyses with binary outcomes. Stat Med.

[80] Sterne JA, Sutton AJ, Ioannidis JP, Terrin N, Jones DR, Lau J, et al. Recommendations for examining and interpreting funnel plot asymmetry in meta-analyses of randomised controlled trials. Bmj. 2011;343:1–8. 10.1136/bmj.d4002 21784880

[81] Munn Z. Software to support the systematic review process: the Joanna Briggs institute system for the unified management. JBI Evidence Synthesis. 2016;14(10):1. 10.11124/JBISRIR-2016-002421 27846109

[82] Rasmussen L, Montgomery P (2018). The prevalence of and factors associated with inclusion of non-English language studies in Campbell systematic reviews: a survey and meta-epidemiological study. Syst Rev.

[83] Aggarwal S, Angus B (2015). Misdiagnosis versus missed diagnosis: diagnosing autism spectrum disorder in adolescents. Australasian Psychiatry.

[84] Lockwood Estrin G, Milner V, Spain D, Happé F, Colvert E. Barriers to autism spectrum disorder diagnosis for young women and girls: A systematic review. Rev J Autism Dev Disord. 2021;8(4):454–70. 10.1007/s40489-020-00225-8 PMC8604819 34868805

[85] Luciano CC, Keller R, Politi P, Aguglia E, Magnano F, Burti L (2014). Misdiagnosis of high function autism spectrum disorders in adults: an Italian case series. Autism Open Access.

[86] Alacha HF, Lefler EK. Negative halo effects in parent ratings of ADHD, ODD, and CD. J Psychopathol Behav Assess. 2021;43(3):1–2.

